# Are parents’ education levels associated with either their oral health knowledge or their children’s oral health behaviors? A survey of 8446 families in Wuhan

**DOI:** 10.1186/s12903-020-01186-4

**Published:** 2020-07-11

**Authors:** Liangwen Chen, Jialan Hong, Dian Xiong, Luyi Zhang, Yuhong Li, Shengfu Huang, Fang Hua

**Affiliations:** 1 Hubei-MOST KLOS & KLOBM, School & Hospital of Stomatology, Wuhan University, No. 237 Luoyu Road, Hongshan District, Wuhan, China; 2 Department of Epidemiology and Public Health, University College London, London, UK; 3 School of Medicine, Wuhan University, Wuhan, China; 4 Wuhan Ulink High School, Wuhan, China; 5 Center for Evidence-Based Stomatology, School & Hospital of Stomatology, Wuhan University, Wuhan, China; 6 Division of Dentistry, School of Medical Sciences, Faculty of Biology, Medicine and Health, The University of Manchester, Manchester Academic Health Science Centre, Manchester, UK

**Keywords:** Pit and fissure sealants, Education level, Mixed dentition, Oral health behavior, Oral health knowledge

## Abstract

**Background:**

Children aged 6–7 years are in the early mixed dentition, which is a period of high prevalence of dental caries and other dental diseases and a critical period for the formation of oral health behaviors. Therefore, good oral hygiene habits of children and oral health knowledge of parents are very important. This study sought to explore the relationship between children’s oral health behaviors, parental oral health knowledge, parental choices of pit and fissure sealants, and parents’ education levels based on a large-scale sample size for the first time, and to compare the influences of parental education levels between parents.

**Methods:**

Families of the first and second graders of primary schools in Wuhan Hongshan District were included in this study. A total of 8446 questionnaires were collected to obtain comprehensive information on children’s oral health behaviors, parents’ oral health knowledge and parents’ pit and fissure sealants-related choices. The relationship between these outcome variables and parents’ education levels were studied using logistic regression analysis and chi-square test.

**Results:**

Parents who reported good educational background had more favorable oral health knowledge than those of other parents, and their children had better oral hygiene behaviors. Four indicators of five measures to children’s oral health behaviors were significantly associated with mother’s education level (*P* < 0.05), and three of them were related to father’s education level (*P* ≤ 0.01). Moreover, seven indicators of eight measures to parents’ oral health knowledge were significantly related to mother’s education level (*P* < 0.05) and four of them were affected by the father’s (P < 0.05). In addition, parents with higher educational attainments paid more attention to the completeness of medical facilities, the environment of dental practice, the distance to treatment sites, and took less concern of children’s willingness when choosing the pit and fissure sealants sites.

**Conclusions:**

In families with children at the early mixed dentition stage, parents with higher education levels tend to have better oral health knowledge and more oral health care needs, such as pit and fissure sealants. In addition, children of parents who have better educated parents tend to perform better oral hygiene practices.

## Background

The results of the fourth National Oral Health Survey of China conducted in 2015–2016 found that the prevalence of permanent dental caries in children aged 12 to 15 years was 41.9%, the mean DFMT was 1.04, and the caries filling rate was 17.5% [1]. These data suggest that the prevention of dental caries in Chinese children is still a problem worthy of attention. This paper will focus on the age group of 6 to 7 years, since children of these ages are in the early mixed dentition that permanent incisors and molars start to erupt and the deciduous teeth remain [2].

In China, 6 to 7 years of age is especially important as it is generally the age at which children begin primary school, which makes reliable samples accessible through the school system. Also, children of these ages are able to acquire knowledge efficiently, which is a critical period for the development of oral hygiene habits [3]. The performance of adequate oral hygiene practices is not only important in the prevention of oral diseases [4], but also in children’s physical and mental health [5]. School-based oral health education programs have been widely conducted in many countries, as these interventions are effective in increasing oral health knowledge, attitudes and behaviors among children [6]. It is notable that parental effects on children’s knowledge, attitudes and behaviors towards oral health are also significant [7].

Previous studies have discussed effects of the socio-economic status of parents on the prevalence of dental caries and oral health behaviors of children [8–10]. Socio-economic status are mainly measured by mother’s education level [11, 12] and household income [13, 14]. The use of education level attempts to capture knowledge and skills-related assets of an individual. Although parental education levels have a certain relation with the indicators of parental occupations and household income [15, 16], their essences are distinct. Household income is a direct and reliable approach to measure household socioeconomic position (SEP).

Pit and fissure sealants (PFS) have been well proven to be effective in preventing dental caries [17–19]. It is suggested by the World Health Organization (WHO) and health guidelines in the majority of countries [20]. Additionally, free PFS treatments have been widely provided by governments over the world [21]. In this study, we collected information on parents’ PFS-related choices and considered it as an extension of parents’ attitudes towards oral health care.

The aim of this study was to explore the relationship between children’s oral health behaviors (COB), parents’ oral health knowledge (POK), parents’ PFS-related choices and parents’ education levels, based on a large sample size for the first time, and to compare the influence of mother and father on COB, POK and PFS-related choices. An additional aim was to identify potential risk factors that might affect children’s oral health.

## Methods

### Study design

Funded by the Hongshan District Government, the Hongshan Longitudinal Study on Pit and Fissure Sealants Application (HoLSPA) project aimed to provide free oral examination and pit and fissure sealants (PFS) for the first and second graders in Hongshan District, Wuhan, China, and at the same time carry out relevant research through oral examinations, data collection and online surveys.

As part of the HoLSPA project, the present study was conducted via an online questionnaire survey targeted at all families of those first and second graders involved in HoLSPA. As determined a priori, responses from 1) single-parent families, 2) parents who had psychiatric or cognitive dysfunctions, 3) parents who did not provide informed consent, or 4) parents who did not fill out the questionnaire completely were excluded from this study.

Electronic questionnaires were sent by the Education Bureau of Hongshan District to parents of 11,000 eligible families through WeChat (a Chinese multi-purpose messaging and social media app) by head teachers. Each family was required to fill out one questionnaire (either by the father or the mother) and return it via WeChat. The survey was completed before the clinical part of HoLSPA project started. In the survey, information on children’s family situation, COB, POK and parents’ PFS-related choice were collected. Full text of the questionnaire can be found in the Additional file 1.

**Fig. 1:**
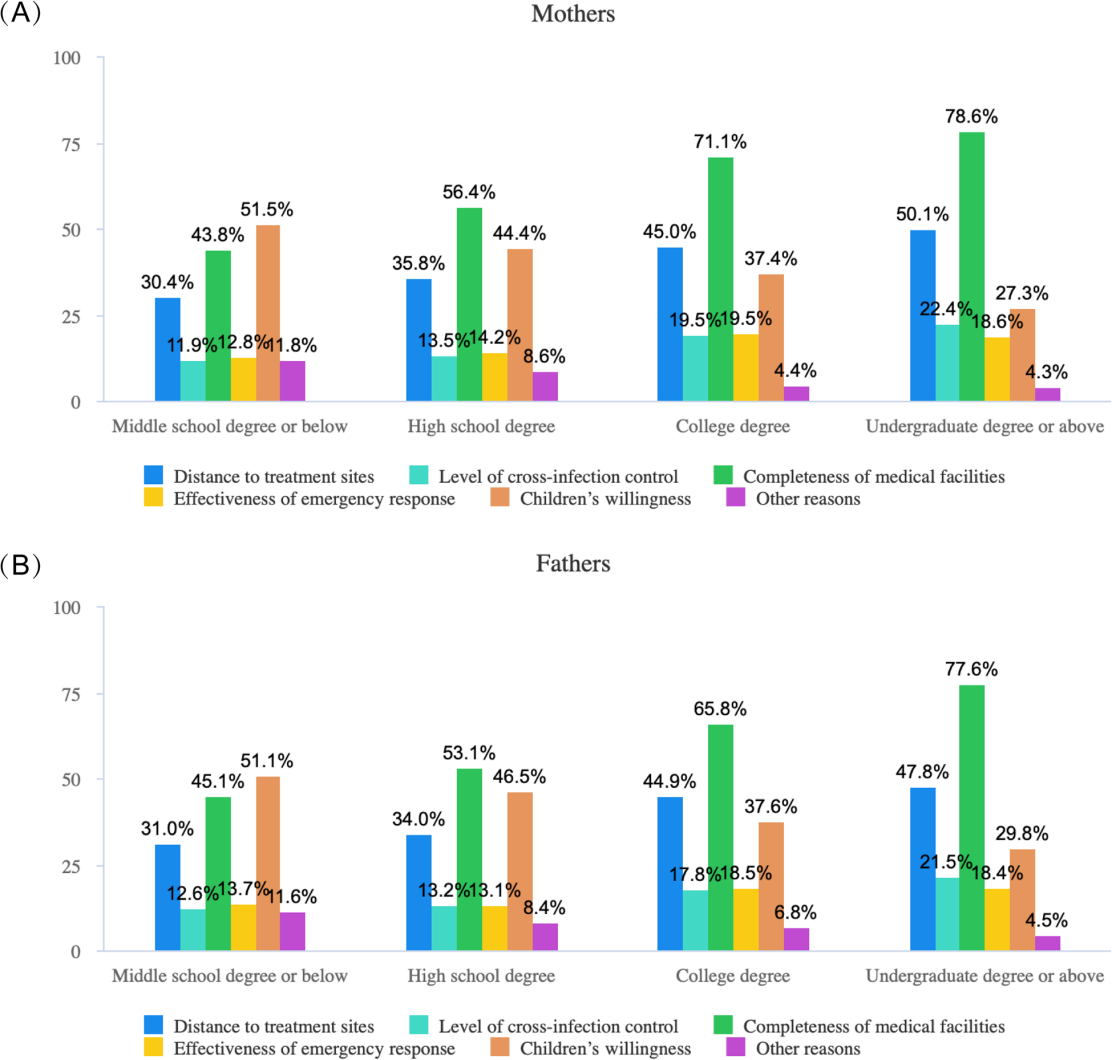
Distribution of the determinants that will be considered by mothers (**a**) and fathers (**b**) when choosing the PFS site

This study was approved by the Medical Ethics Committee of Hospital of Stomatology, Wuhan University (2018. B13). Written informed consents were obtained from all participants of this study.

### Questionnaire

#### Family characteristics

The family characteristics of the respondents included the number of children in the family, father’s education level and mother’s education level. Father’s and mother’s educational attainments were grouped into four categories: “Middle school degree or below”, “High school degree”, “College degree”, “Undergraduate degree or above”.

#### Children’s oral health behaviors

Five questions were used to analyze the relationship between COB and parents’ education levels. The measures of COB included: (1) Does the child usually brush his/her teeth? (2) Does the child brush his/her teeth at least twice a day? (3) Has the child ever visited a dentist? (4) Was the last dental visit within the past 12 months? (5) The main reason for the last dental visit. The first four questions required a ‘yes’ or ‘no’ answer, and two options of the last question were “treatment” and “consultation”.

#### Parents’ oral health knowledge

The relationships between POK and parental education levels were examined. POK was measured by the response to following true-false statements: (1) Gingival bleeding is normal when brushing teeth; (2) Gingivitis is caused by bacterial infection; (3) Tooth-brushing is helpful in preventing gingivitis; (4) Dental caries is mainly caused by pathogenic bacteria; (5) Sugar intake is associated with dental caries; (6) Fluoride protects teeth from decay; (7) Pit and fissure sealants help in preventing dental caries; (8) Oral health is essential to general health. The answers were grouped into two categories: “correct” and “wrong and do not know”.

#### Parents’ pit and fissure sealants (PFS) related choices

Three questions explored the relationship between parents’ opinions of PFS and their education. Questions on parents’ opinions of PFS included: (1) Have you ever heard of PFS? (“yes” or “no”); (2) Where would you prefer to have PFS? (“dental hospital” or “school”); (3) What are the determinants of choosing PFS location? (“distance to treatment sites”, “level of cross-infection control”, “completeness of medical facilities”, “effectiveness of emergency response”, “children’s willingness” and “other reasons”).

### Questionnaire validation

Since the HoLSPA project was free, families from all social backgrounds could participate in the project. The HoLSPA was supervised by Education Bureau and the Health Commission of Hongshan District, which guaranteed an adequate response rate. The questionnaire questions mainly came from the Fourth National Oral Survey of China (2015–2016), which was developed according to the WHO guidelines [22] by Chinese Stomatological Association. A class of students in the Wuhan Primary School was randomly selected for pilot study one week prior to the launch of the main trial to test the repeatability of the questionnaire. There were no significant differences between the results of the pilot study and the main study, suggesting the repeatability of this questionnaire was convincing.

### Statistical analysis

We first assessed the crude associations between family characteristics, COB, POK, parents’ PFS-related choices and parental education levels using Chi-square tests. Then, logistic regression analyses were performed to examine the association between COB and parents’ education levels. Associations between children’s oral health and parents’ education levels were not always consistent for the father and the mother [23]. Thus, we conducted univariate analyses to investigate the association between COB and mother’s and father’s education level independently, and then assessed the educational effects of both parents simultaneously in the multivariate model.

Next, we examined the association between POK and parental education levels using logistic regression analysis. Finally, logistic regression analyses were performed to examine the association between parental education and their understanding of PFS as a proxy of parents’ choices of oral health care. We stratified all analysis by parent’s gender to control the confounding effect of gender. EpiData 3.0 was used for data entry and SPSS 25.0 was used for data analysis. A significant level of 0.05 was used to determine statistical significance. Additionally, histograms were drawn by SPSSAU to demonstrate the relationship between the determinants of PFS location choice and parents’ education separately.

## Results

### Characteristics of the participants

Our questionnaire was sent to a total of 11,000 families, 2459 of which did not participate in this survey. Among the 8541 responses that we received, 95 were either incomplete or from single-parent families and were therefore excluded. Finally, 8446 eligible responses from 8446 families (with children from 43 primary schools in Hongshan District, Wuhan) were included in this analysis, resulting in a response rate of 76.78% (8446/11000).

The distribution of family characteristics, COB, POK, and parents’ PFS-related choices by parental education levels were presented in Table 1. The number of families that had only one child (50.82%) was slightly higher than those with two or more children (49.18%). As shown in Table 1, parents with a lower level of education were more likely to have more than one child. According to the results of the chi-square test, the responses of all questions were significantly related to both parents’ academic backgrounds (*P* < 0.001). Overall, parents in higher education groups had better oral health knowledge and their children reported more favorable behaviors than their counterparts.

**Table 1:** Chi-square tests of the response to each question of participants’ families (*n* = 8446)

	Row total n(%)	Mother’s education level n(%) (***n*** = 8385)					Father’s education level n(%) (***n*** = 8397)				
		Middle school or below	High school	College	Undergraduate or above	x^**2**^-test (P)	Middle school or below	High school	College	Undergraduate or above	x^**2**^-test (P)
**Family characteristics**											
Number of children [one (omitted) vs. two or more] *n* = 8446	4154 (49.18)	**1544(72.28)**	**1154(52.57)**	**608(36.89)**	**843(34.52)**	**< 0.001**	**1279(71.93)**	**1132(54.95)**	**605(39.52)**	**1115(36.82)**	**< 0.001**
**Oral health-related behaviors of children**											
Brushing teeth [no (omitted) vs. yes] *n* = 8446	8120 (96.14)	**2004(93.82)**	**2109(96.08)**	**1615(98.00)**	**2371(97.09)**	**< 0.001**	**1668(93.81)**	**1979(96.07)**	**1484(96.93)**	**2946(97.29)**	**< 0.001**
Brushing frequency [once (omitted) vs. twice or more] *n* = 8120	4565 (56.22)	**747(37.28)**	**1095(51.92)**	**1012(62.66)**	**1703(71.83)**	**< 0.001**	**602(36.09)**	**968(48.91)**	**914(61.59)**	**2054(69.72)**	**< 0.001**
Dental visit [no (omitted) vs. yes] n = 8446	5898 (69.83)	**1284(60.11)**	**1444(65.79)**	**1237(75.06)**	**1920(78.62)**	**< 0.001**	**1039(58.44)**	**1365(66.26)**	**1116(72.89)**	**2343(77.38)**	**< 0.001**
Dental visit in past 12 months [no (omitted) vs. yes] *n* = 5898	4233 (71.77)	**873(67.99)**	**1023(70.84)**	**885(71.54)**	**1445(75.26)**	**< 0.001**	**708(68.14)**	**939(68.79)**	**794(71.15)**	**1772(75.63)**	**< 0.001**
Reasons for dental visit [consultation (omitted) vs. treatment] *n* = 4200	1839 (43.79)	**284(33.29)**	**426(41.72)**	**414(46.99)**	**713(49.55)**	**< 0.001**	**229(32.90)**	**389(41.87)**	**361(45.64)**	**851(48.22)**	**< 0.001**
**Oral health knowledge literacy of parents**											
Gingival bleeding is normal [correct (omitted) vs. wrong] *n* = 8318	6167 (74.14)	**1783(86.01)**	**1964(90.59)**	**1487(91.12)**	**2245(92.77)**	**< 0.001**	**1476(85.22)**	**1833(90.56)**	**1392(91.70)**	**2761(92.16)**	**< 0.001**
Gingivitis is caused by bacteria [wrong (omitted) vs. correct] *n* = 8329	7744 (92.98)	**1887(90.29)**	**2011(92.80)**	**1531(94.04)**	**2292(94.71)**	**< 0.001**	**1586(90.89)**	**1868(92.20)**	**1413(93.08)**	**2842(94.95)**	**< 0.001**
Tooth-brushing prevent gingivitis [wrong (omitted) vs. correct] *n* = 8309	7198 (86.63)	**1660(80.15)**	**1843(85.17)**	**1443(88.64)**	**2232(92.19)**	**< 0.001**	**1417(81.86)**	**1681(83.14)**	**1320(87.13)**	**2741(91.58)**	**< 0.001**
Dental caries is caused by bacteria [wrong (omitted) vs. correct] *n* = 8330	6941 (83.33)	**1585(76.13)**	**1770(81.64)**	**1401(85.85)**	**2167(89.43)**	**< 0.001**	**1363(78.42)**	**1611(79.59)**	**1282(84.23)**	**2654(88.47)**	**< 0.001**
Sugar intake can cause dental caries [wrong (omitted) vs. correct] *n* = 8342	7118 (85.33)	**1722(82.31)**	**1801(82.92)**	**1407(86.27)**	**2166(89.43)**	**< 0.001**	**1448(83.08)**	**1679(82.51)**	**1299(85.40)**	**2657(88.68)**	**< 0.001**
Fluoride protects teeth from decay (wrong vs. correct)1 = Correct *n* = 8296	5296 (63.84)	**1060(51.23)**	**1240(57.46)**	**1050(64.54)**	**1848(76.40)**	**< 0.001**	**902(52.17)**	**1140(56.66)**	**945(62.38)**	**2197(73.40)**	**< 0.001**
PFS help in preventing caries (wrong vs. correct)1 = Correct *n* = 8325	7373 (88.56)	**1690(81.13)**	**1902(87.81)**	**1466(90.22)**	**2297(94.68)**	**< 0.001**	**1421(81.71)**	**1725(85.31)**	**1371(90.38)**	**2823(94.13)**	**< 0.001**
Oral health is essential to general health [wrong (omitted) vs. correct] *n* = 8334	7605 (91.25)	**1766(84.46)**	**1954(90.17)**	**1530(93.87)**	**2332(96.32)**	**< 0.001**	**1481(85.02)**	**1799(88.80)**	**1404(92.43)**	**2882(96.10)**	**< 0.001**
**PFS-related choices of parents**											
Ever heard of PFS [no (omitted) vs. yes] n = 8446	6867 (81.30)	**1495(69.99)**	**1734(79.00)**	**1414(85.80)**	**2204(90.25)**	**< 0.001**	**1229(69.12)**	**1601(77.72)**	**1285(83.93)**	**2717(89.73)**	**< 0.001**
Preference of PFS site [school (omitted) vs. hospital] n = 8446	5739 (67.95)	**1117(52.29)**	**1388(63.23)**	**1209(73.36)**	**2013(82.43)**	**< 0.001**	**905(50.90)**	**1266(61.46)**	**1064(69.50)**	**2476(81.77)**	**< 0.001**
**Total**		2100(25.04)	2195(26.18)	1648(19.65)	2442(29.12)		1778(21.12)	2060(24.53)	1531(18.23)	3028(36.06)	

Significant associations (*P* < 0.05) are in bold

### Children’s oral health behaviors

Table 2 demonstrated logistic regression results of the effects of mother’s and father’s educational attainments on COB. In the univariate model, mother’s and father’s education levels were all associated with children’s tooth-brushing behaviors. When adjusted for education level of counterpart parent, children’s tooth-brushing behavior can be predicted by mother’s education level rather than father’s education level. Participants whose mother had college degree (Odds Ratio [OR] = 2.62, 95% Confidential Interval [CI] = 1.57–4.35) and undergraduate degree or above (OR = 1.72, 95%CI = 1.03–2.85) were more likely to brush their teeth than those whose mother with middle school education or below. A clear increased gradient in the effects of parental education levels on tooth-brushing frequency was found, showing that children whose parents with higher education levels were more likely to brush their teeth twice a day or more. For example, children whose mother with university degree were 2.47 (95%CI = 2.01–3.02) more likely to brush teeth at least twice a day than those whose mother in the lowest education group, and the effect size of father’s education level (OR = 2.05, 95%CI = 1.68–2.51) was slightly smaller than that of mother’s. Similarly, the proportion of children ever had dental visit was steadily increasing as the parental education levels rose. The odds of children ever had dental visit was 1.79 (95%CI = 2.45–3.32) times and 1.52 (95%CI = 1.24–1.87) times higher among mothers with university degrees and fathers with university degrees than their counterparts with middle school education or lower, respectively. When further exploring the last dental visit, participants whose father ever attended university were 1.41 (95CI% = 1.09–1.83) times more likely to had dental visit in the past 12 months than those whose father with lowest educational background. Additionally, those whose mother with higher education levels were more likely to visit dentists for dental treatment (OR = 1.28, 95%CI = 1.02–1.61 for high school degree; OR = 1.61, 95%CI = 1.22–2.11 for college degree; OR = 1.70, 95%CI = 1.35–2.37 for university degree or above). The reason for dental visit was associated with father’s education level in the univariate model, however, this association was eliminated after adjusted for mother’s education level.

**Table 2:** Univariable and multivariable logistic regression derived coefficients (OR), 95% confidence intervals and P value, for the association between children’s oral health behavior and parents’ education levels

Oral health related habits	Educational level	Univariable						Multivariable					
		Mother			Father			Mother			Father		
		OR	95%CI	P	OR	95%CI	P	OR	95%CI	P	OR	95%CI	P
Brushing teeth	Middle school or below	Reference			Reference			Reference			Reference		
(0 = “no”,	High school	**1.62**	**1.22–2.13**	**0.001**	**1.61**	**1.20–2.16**	**0.001**	1.36	0.96–1.91	0.080	1.31	0.93–1.85	0.118
1 = “yes”)	College	**3.22**	**2.10–4.75**	**< 0.001**	**2.08**	**1.47–2.95**	**< 0.001**	**2.62**	**1.57–4.35**	**0.037**	1.25	0.79–1.97	0.344
	Undergraduate or above	**2.20**	**2.20–1.64**	**< 0.001**	**2.37**	**1.77–3.17**	**< 0.001**	**1.72**	**1.03–2.85**	**< 0.001**	1.42	0.86–2.34	0.176
Brushing frequency	Middle school or below	Reference			Reference			Reference			Reference		
(0 = “≤ once”,	High school	**1.82**	**1.60–2.06**	**< 0.001**	**1.70**	**1.48–1.94**	**< 0.001**	**1.43**	**1.22–1.65**	**< 0.001**	**1.37**	**1.17–1.60**	**< 0.001**
1 = “≥ twice”)	College	**2,83**	**2.47–3.23**	**< 0.001**	**2.84**	**2.46–3.28**	**< 0.001**	**1.77**	**1.47–2.14**	**< 0.001**	**1.81**	**1.49–2.19**	**< 0.001**
	Undergraduate or above	**4.29**	**3.78–4.87**	**< 0.001**	**4.08**	**3.59–4.63**	**< 0.001**	**2.47**	**2.01–3.02**	**< 0.001**	**2.05**	**1.68–2.51**	**< 0.001**
Dental Visit	Middle school or below	Reference			Reference			Reference			Reference		
(0 = “no”,	High school	**1.28**	**1.13–1.44**	**< 0.001**	**1.40**	**1.22–1.59**	**< 0.001**	1.08	0.93–1.26	0.311	**1.30**	**1.11–1.51**	**< 0.001**
1 = “yes”)	College	**2.00**	**1.73–2.30**	**< 0.001**	**1.91**	**1.65–2.22**	**< 0.001**	**1.52**	**1.25–1.86**	**< 0.001**	**1.44**	**1.18–1.75**	**< 0.001**
	Undergraduate or above	**2.44**	**2.14–2.78**	**< 0.001**	**2.43**	**2.14–2.76**	**< 0.001**	**1.79**	**1.45–2.21**	**< 0.001**	**1.52**	**1.24–1.87**	**< 0.001**
Dental visit in past 12 months	Middle school or below	Reference			Reference			Reference			Reference		
(0 = “no”,	High school	1.14	0.97–1.35	0.106	1.03	0.87–1.23	0.734	1.09	0.89–1.33	0.394	0.99	0.81–1.21	0.915
1 = “yes”)	College	1.18	1.00–1.40	0.052	1.15	0.96–1.39	0.130	0.99	0.78–1.26	0.913	1.14	0.89–1.46	0.301
	Undergraduate or above	**1.43**	**1.23–1.78**	**< 0.001**	**1.45**	**1.24–1.70**	**< 0.001**	1.06	0.82–1.37	0.638	**1.41**	**1.09–1.83**	**0.010**
Dental visit reason	Middle school or below	Reference			Reference			Reference			Reference		
(0 = “consultation”,	High school	**1.43**	**1.19–1.73**	**< 0.001**	**1.47**	**1.20–1.80**	**< 0.001**	**1.28**	**1.02–1.61**	**0.034**	1.26	0.99–1.60	0.060
1 = “treatment”)	College	**1.78**	**1.46–2.16**	**< 0.001**	**1.71**	**1.39–2.12**	**< 0.001**	**1.61**	**1.22–2.11**	**0.001**	1.21	0.91–1.60	0.194
	Undergraduate or above	**1.97**	**1.65–2.35**	**< 0.001**	**1.90**	**1.58–2.28**	**< 0.001**	**1.70**	**1.35–2.37**	**< 0.001**	1.19	0.89–1.59	0.246

Significant associations (*P* < 0.05) are in bold

### Parents’ oral health knowledge

Table 3 showed the relationship between parental education levels and correctness rates to true-false oral health-related statements. The multivariate logistic regression results showed that correctness rates of seven oral health-related statements were significantly related to mother’s academic qualification, and correctness rates of four statements were significantly related to father’s education level.

**Table 3:** Univariable and multivariable logistic regression derived coefficients (OR), 95% confidence intervals and P value, for the association between parents’ oral health knowledge and parents’ education levels

Oral health	Educational level	Univariable						Multivariable					
		Mother			Father			Mother			Father		
		OR	95%CI	P	OR	95%CI	P	OR	95%CI	P	OR	95%CI	P
Gingival bleeding is normal when brushing teeth (1 = “wrong”)	Middle school or below	Reference			Reference			Reference			Reference		
	High school	**1.40**	**1.22–1.59**	**< 0.001**	**1.36**	**1.18–1.56**	**< 0.001**	**1.25**	**1.07–1.47**	0.006	**1.19**	**1.01–1.40**	**0.040**
	College	**1.56**	**1.35–1.81**	**< 0.001**	**1.69**	**1.44–1.97**	**< 0.001**	**1.39**	**1.13–1.71**	0.002	**1.26**	**1.03–1.55**	**0.027**
	Undergraduate or above	**2.21**	**1.93–2.13**	**< 0.001**	**1.89**	**1.65–2.15**	**< 0.001**	**2.05**	**1.64–2.56**	**< 0.001**	1.12	0.90–1.39	0.300
Gingivitis is caused by bacterial infection (1 = “correct”)	Middle school or below	Reference			Reference			Reference			Reference		
	High school	**1.39**	**1.12–1.72**	**0.003**	1.19	0.94–1.49	0.148	1.28	0.98–1.67	0.068	1.03	0.79–1.35	0.812
	College	**1.70**	**1.32–2.18**	**< 0.001**	**1.35**	**1.04–1.74**	**0.022**	1.40	0.99–1.99	0.058	1.05	0.75–1.49	0.769
	Undergraduate or above	**1.93**	**1.53–2.42**	**< 0.001**	**1.89**	**1.50–2.38**	**< 0.001**	1.40	0.96–2.04	0.085	1.43	0.98–2.09	0.064
Tooth-brushing helps in preventing gingivitis (1 = “correct”)	Middle school or below	Reference			Reference			Reference			Reference		
	High school	**1.42**	**1.21–1.67**	**< 0.001**	1.09	0.92–1.29	0.305	**1.47**	**1.21–1.78**	**< 0.001**	0.87	0.71–1.05	0.151
	College	**1.93**	**1.60–2.33**	**< 0.001**	**1.50**	**1.24–1.82**	**< 0.001**	**1.79**	**1.39–2.32**	**< 0.001**	0.94	0.73–1.21	0.626
	Undergraduate or above	**2.92**	**2.43–3.51**	**< 0.001**	**2.41**	**2.02–2.88**	**< 0.001**	**2.42**	**1.81–3.23**	**< 0.001**	1.21	0.91–1.61	0.180
Dental caries is caused by bacteria (1 = “correct”)	Middle school or below	**Reference**			Reference			Reference			Reference		
	High school	**1.39**	**1.20–1.62**	**< 0.001**	1.07	0.92–1.26	0.379	**1.48**	**1.24–1.77**	**< 0.001**	0.85	0.71–1.02	0.850
	College	**1.90**	**1.60–2.26**	**< 0.001**	**1.47**	**1.23–1.76**	**< 0.001**	**1.88**	**1.48–2.39**	**< 0.001**	0.90	0.71–1.14	0.901
	Undergraduate or above	**2.65**	**2.25–3.13**	**< 0.001**	**2.11**	**1.80–2.48**	**< 0.001**	**2.46**	**1.89–3.20**	**< 0.001**	1.05	0.81–1.36	1.048
Sugar intake can cause dental caries (1 = “correct”)	Middle school or below	Reference			Reference			Reference			Reference		
	High school	1.04	0.89–1.22	0.602	0.96	0.81–1.14	0.644	1.08	0.89–1.31	0.429	0.90	0.74–1.10	0.901
	College	**1.35**	**1.13–1.62**	**0.001**	1.19	0.99–1.44	0.069	**1.29**	**1.00–1.66**	**0.044**	0.97	0.75–1.24	0.968
	Undergraduate or above	**1.82**	**1.53–2.16**	**< 0.001**	**1.60**	**1.35–1.89**	**< 0.001**	**1.65**	**1.26–2.17**	**< 0.001**	1.09	0.84–1.43	1.093
Fluoride protects teeth from decay (1 = “correct”)	Middle school or below	Reference			Reference			Reference			Reference		
	High school	**1.29**	**1.14–1.45**	**< 0.001**	**1.20**	**1.05–1.36**	**0.006**	**1.21**	**1.05–1.41**	**0.010**	1.05	0.90–1.22	0.558
	College	**1.73**	**1.52–1.98**	**< 0.001**	**1.52**	**1.32–1.75**	**< 0.001**	**1.53**	**1.27–1.84**	**< 0.001**	1.06	0.88–1.28	0.561
	Undergraduate or above	**3.08**	**2.71–3.50**	**< 0.001**	**2.533**	**2.24–2.87**	**< 0.001**	**2.49**	**2.03–3.05**	**< 0.001**	**1.30**	**1.06–1.58**	**0.012**
PFS help in preventing dental caries (1 = “correct”)	Middle school or below	Reference			Reference			Reference			Reference		
	High school	**1.68**	**1.42–1.98**	**< 0.001**	**1.30**	**1.09–1.55**	**0.003**	**1.42**	**1.16–1.74**	**0.001**	1.08	0.88–1.32	0.486
	College	**2.14**	**1.76–2.61**	**< 0.001**	**2.10**	**1.70–2.59**	**< 0.001**	**1.38**	**1.05–1.81**	**0.022**	**1.53**	**1.16–2.02**	**0.003**
	Undergraduate or above	**4.14**	**3.36–5.10**	**< 0.001**	**3.59**	**2.95–4.36**	**< 0.001**	**2.25**	**1.63–3.10**	**< 0.001**	**2.03**	**1.49–2.76**	**< 0.001**
Oral health is essential to general health (1 = “correct”)	Middle school or below	Reference			Reference			Reference			Reference		
	High school	**1.69**	**1.40–2.03**	**< 0.001**	**1.40**	**1.15–1.69**	**0.001**	**1.47**	**1.18–1.84**	**0.001**	1.10	0.88–1.38	0.395
	College	**2.82**	**2.23–3.56**	**< 0.001**	**2.15**	**1.71–2.71**	**< 0.001**	**1.97**	**1.43–2.70**	**< 0.001**	1.29	0.95–1.74	0.103
	Undergraduate or above	**4.82**	**3.78–6.15**	**< 0.001**	**4.34**	**3.46–5.45**	**< 0.001**	**2.72**	**1.88–3.93**	**< 0.001**	**2.03**	**1.43–2.89**	**< 0.001**

Significant associations (*P* < 0.05) are in bold

The correctness rates of answers to “bleeding gums are normal when brushing teeth”, “tooth-brushing is helpful in preventing gingivitis”, “dental caries is mainly caused by pathogenic bacteria”, “sugar intake is associated with dental caries”, “fluoride protects teeth from decay”, “PFS help in preventing dental caries” and “oral diseases is essential to general health” were significantly lower among mothers with lower education than those of higher education. Regarding to fathers’ responses, the correctness rates of answers to “fluoride protects teeth from decay”, “PFS help in preventing dental caries” and “oral diseases is essential to general health” were significantly higher among fathers with undergraduate degrees or above than fathers with lowest educational background (OR = 1.30, 95%CI = 1.06–1.58; OR = 2.03, 95%CI = 1.49–2.76; OR = 2.03, 95%CI = 1.43–2.89, respectively). Moreover, fathers ever attended high schools or colleges were 1.19 (95%CI = 1.01–1.40) and 1.26 (95%CI = 1.03–1.55) times more likely to report correct answer to “bleeding gums are normal when brushing teeth” than those with lowest level of education.

### Parents’ pit and fissure sealing related choices

Table 4 presented the results of logistic regression analysis between parents’ education levels and their PFS-related choices. The odds of PFS awareness rate increased with both mother’s and father’s education level. Compared with mothers attended nine-year education or less, having high school, college and undergraduate degrees or above yielded ORs of 1.29 (95% CI = 1.09–1.53), 1.65 (95% CI = 1.31–2.08), and 2.15 (95% CI = 1.67–2.78), respectively. The effect size of father’s education level on PFS awareness was similar to mother’s.

**Table 4:** Univariable and multivariable logistic regression derived coefficients (OR), 95% confidence intervals and P value, for the association between parents’ PFS related choices and their education levels

Oral health related habits	Educational level	Univariable						Multivariable					
		Mother			Father			Mother			Father		
		OR	95%CI	P	OR	95%CI	P	OR	95%CI	P	OR	95%CI	P
Ever heard of FPS	Middle school or below	Reference			Reference			Reference			Reference		
(0 = no,	High school	**1.61**	**1.41–1.85**	**< 0.001**	**1.56**	**1.35–1.80**	**< 0.001**	**1.29**	**1.09–1.53**	**0.003**	**1.21**	**1.11–1.55**	**0.003**
1 = yes)	College	**2.59**	**2.19–3.06**	**< 0.001**	**2.33**	**1.97–2.76**	**< 0.001**	**1.65**	**1.31–2.08**	**< 0.001**	**1.59**	**1.27–1.99**	**< 0.001**
	Undergraduate or above	**3.97**	**3.38–4.67**	**< 0.001**	**3.90**	**3.34–4.56**	**< 0.001**	**2.15**	**1.67–2.78**	**< 0.001**	**2.15**	**1.68–2.75**	**< 0.001**
Preference of PFS site	Middle school or below	Reference			Reference			Reference			Reference		
(0 = school,	High school	**1.60**	**1.39–1.77**	**< 0.001**	**1.54**	**1.35–1.75**	**< 0.001**	**1.23**	**1.07–1.43**	**0.005**	**1.33**	**1.15–1.55**	**< 0.001**
1 = hospital)	College	**2.51**	**2.19–2.89**	**< 0.001**	**2.20**	**1.91–2.54**	**< 0.001**	**1.56**	**1.29–1.89**	**< 0.001**	**1.54**	**1.28–1.87**	**< 0.001**
	Undergraduate or above	**4.28**	**3.74–4.90**	**< 0.001**	**4.33**	**3.80–4.93**	**< 0.001**	**2.14**	**1.73–2.64**	**< 0.001**	**2.43**	**1.97–2.98**	**< 0.001**

Significant associations (*P* < 0.05) are in bold

Regarding to the preference of PFS location for children, the proportion of choosing dental hospitals increased as the higher academic qualifications gained among both mothers and fathers. The effect size of education levels on PFS location preference was stronger among fathers than mothers.

Figure 1 A and B presented the distribution of parental education levels and conditions that will be considered when selecting the location of PFS. The trends shown in the two figures were generally alike, showing that the proportions of parents who considered “completeness of medical facilities” and “distance to PFS sites” rose with increase in their education levels. In contrast, the proportion of parents who listened to “children’s willingness” decreased with increase academic achievements

## Discussion

This is the first study carried out in China to assess the relationship between COB, POK, parents’ choice of PFS and parental education levels among first and second graders’ families.

This study suggested that parental education levels were associated with oral health-related issues. Firstly, children were more likely to practice oral hygiene care with the increase of parental education levels, specifically, children from well-educated families were more likely to brush teeth, brush teeth more often, visit the dentist more frequent, and have regular dental check. Secondly, oral health literacy was increased with education levels of parents. Thirdly, parents with higher educational background not only had a better understanding of PFS, but also placed greater emphasis on PFS practice environment, completeness of medical facilities and distance to PFS sites, while less consideration was given to children’s willingness.

When it comes to COB, mother’s education level significantly affected 4 of 5 indicators, while father’s education level only affected 3 indicators. Regarding to POK, mother’s education level was significantly associated with correctness rates of 7 of 8 statements, 3 more than that of father’s. Moreover, the PFS-related choices were significantly related to both mother’s and father’s education levels. It appeared that the mother’s educational background may play a more important role than father’s in the development of family’s oral health knowledge and behaviors. This finding is consistent with the results of a study in a group of Latinos, which suggested that mothers were primarily responsible for brushing children’s teeth, overseeing the child’s diet and seeking dental care for their child; while fathers believed that managing financial issues for health care was their primarily responsibility [24]. Folayan et al. [25] also proved that mothers’ oral health behaviors were significant predictors of their children’s oral health behaviors.

In agreement with the findings of Schwendicke et al., [26] that people with lower own or parental education levels would have poorer health literacy, poorer dietary and oral health behaviors, our study found that parents with lower academic backgrounds had poorer oral health knowledge, which in turn leaded to poorer oral health behaviors. A Belgian study found that 5-year-old children whose mothers with higher education levels were more likely to consume less sugary drinks, brush teeth more frequently, have more dental visits, and have lower prevalence of dental caries [27]. Similar pattern has also been reported previously in a study regarding to dental caries, suggesting that mother’s education level was the most important index that affect dental caries than household income and parents’ occupations [13]. In line with the study conducted by Franzman et al. [28] showing that daily brushing frequency of a group of American children was positively associated with both mother’s and father’s education levels, our findings also demonstrated that both mother’s and father’s education levels had significant influences on COB.

Nunez et al. [29] and Camargo et al. [30] both suggested that higher parental education levels were associated with more frequent use of dental services, which was revalidated in our study. Interestingly, our findings showed that higher educated parents were more likely to visit dentist for the purpose of dental treatment rather than dental consultation. This might because of the higher internet use for health information acquisition among higher educated groups when encountering health problems.

AI Agili and Griffin [31] claimed that higher parental education and family income were associated with higher sealants prevalence. Our research added that higher educated parents paid more attention to distance to PFS sites, and completeness of medical facilities, and PFS practice environment. Interestingly, they took less account to children’s willingness than the lower educated counterparts when choosing the PFS sites. It might because that higher educated parents were more uncompromising and autocratic in decision making.

The limitation of this study was that the survey was carried out in Hongshan District, Wuhan, where 38 high-level colleges and universities are located in this district. Because of the great density and quality of educational resources, local parents’ education levels are generally higher than most cities in China. Therefore, our sample cannot represent the Chinese population to a certain degree. Another limitation was the lack of information on sociodemographic variables (e.g. household income [13, 14] and living conditions [32–34]), as such information were not included in our questionnaire and therefore not adjusted in our analysis. Further studies are recommended to verify the results of our study on the basis of adjustment for the above-mentioned factors.

## Conclusion

In families with children at the early mixed dentition stage, parents with higher education levels tend to have better oral health knowledge and have more oral health care needs, such as pit and fissure sealants. Children of parents who have higher educational background tend to perform better oral hygiene practices.

## Supplementary information

**Additional file 1.** Questionnaire for Wuhan Hongshan District Primary School Students’ Families.

## Data Availability

The parents’ data will not be shared. However, the datasets used and analyzed during the current study are available from the corresponding authors on reasonable request.

## Abbreviations

COB: Children’s oral health behaviors
POK: Parents’ oral health knowledge
PFS: Pit and fissure sealing

## References

[1] Quan JK, Wang XZ, Sun XY, Yuan C, Liu XN, Wang X, Feng XP, Tai BJ, Hu Y, Lin HC (2018). Permanent teeth caries status of 12- to 15-year-olds in China: findings from the 4th National Oral Health Survey. Chin J Dent Res.

[2] Ahiko N, Baba Y, Tsuji M, Horikawa R, Moriyama K (2019). Investigation of maxillofacial morphology and oral characteristics with turner syndrome and early mixed dentition. Congenit Anom (Kyoto).

[3] Kaminska A, Szalewski L, Batkowska J, Wallner J, Wallner E, Szabelska A, Borowicz J (2016). The dependence of dental caries on oral hygiene habits in preschool children from urban and rural areas in Poland. Ann Agric Environ Med.

[4] De David SC, Mario TG, De Freitas GC, Kantorski KZ, Wikesjo UME, Moreira CHC (2018). Correlation between plaque control and gingival health using short and extended oral hygiene intervals. Clin Oral Investig.

[5] Kisely S (2016). No mental Health without Oral Health. Can J Psychiatr.

[6] Angelopoulou MV, Kavvadia K, Taoufik K, Oulis CJ (2015). Comparative clinical study testing the effectiveness of school based oral health education using experiential learning or traditional lecturing in 10 year-old children. BMC Oral Health.

[7] Ji Y, Zhang Y, Wang Y, Chang C (2016). Association between family factors and children's oral health behaviors--a cross-sectional comparative study of permanent resident and migrant children in large cities in China. Community Dent Oral Epidemiol.

[8] Pieper K, Dressler S, Heinzel-Gutenbrunner M, Neuhauser A, Krecker M, Wunderlich K, Jablonski-Momeni A (2012). The influence of social status on pre-school children's eating habits, caries experience and caries prevention behavior. Int J Public Health.

[9] Kumar S, Tadakamadla J, Kroon J, Johnson NW (2016). Impact of parent-related factors on dental caries in the permanent dentition of 6-12-year-old children: a systematic review. J Dent.

[10] Boyce WT, Den Besten PK, Stamperdahl J, Zhan L, Jiang Y, Adler NE, Featherstone JD (2010). Social inequalities in childhood dental caries: the convergent roles of stress, bacteria and disadvantage. Soc Sci Med.

[11] Veerasamy A, Kirk R, Gage J (2016). Epidemiology of dental caries among adolescents in Tamil Nadu, , India. Int Dent J.

[12] Pizzo G, Piscopo MR, Matranga D, Luparello M, Pizzo I, Giuliana G (2010). Prevalence and socio-behavioral determinants of dental caries in Sicilian schoolchildren. Med Sci Monitor.

[13] van der Tas JT, Kragt L, Elfrink MEC, Bertens LCM, Jaddoe VWV, Moll HA, Ongkosuwito EM, Wolvius EB (2017). Social inequalities and dental caries in six-year-old children from the Netherlands. J Dent.

[14] Vettore MV, Ahmad SFH, Machuca C, Fontanini H (2019). Socio-economic status, social support, social network, dental status, and oral health reported outcomes in adolescents. Eur J Oral Sci.

[15] Hoque KE, Hoque KF (2018). R APT: relationships between parents' academic backgrounds and incomes and building students' healthy eating habits. PeerJ.

[16] Kerkeni E, Monastiri K, Saket B, Rudan D, Zgaga L, Ben Cheikh H (2006). Association among education level, occupation status, and consanguinity in Tunisia and Croatia. Croat Med J.

[17] Chestnutt IG, Hutchings S, Playle R, Morgan-Trimmer S, Fitzsimmons D, Aawar N, Angel L, Derrick S, Drew C, Hoddell C (2017). Seal or varnish? A randomised controlled trial to determine the relative cost and effectiveness of pit and fissure sealant and fluoride varnish in preventing dental decay. Health Technol Assess.

[18] Khouja T, Smith KJ (2018). Cost-effectiveness analysis of two caries prevention methods in the first permanent molar in children. J Public Health Dent.

[19] Chestnutt IG, Playle R, Hutchings S, Morgan-Trimmer S, Fitzsimmons D, Aawar N, Angel L, Derrick S, Drew C, Hoddell C (2017). Fissure seal or fluoride varnish? A randomized trial of relative effectiveness. J Dent Res.

[20] Baldini V, Tagliaferro EP, Ambrosano GM, Meneghim Mde C, Pereira AC (2011). Use of occlusal sealant in a community program and caries incidence in high- and low-risk children. J Appl Oral Sci.

[21] Corbin SB, Kleinman DV, Lane JM (1985). New opportunities for enhancing oral health: moving toward the 1990 objectives for the nation. Public Health Rep.

[22] Petersen PE, Baez RJ, World Health O (2013). Oral health surveys: basic methods, 5th ed edn.

[23] Blumer S, Ratson T, Peretz B, Dagon N (2018). Parents' attitude towards the use of fluorides and fissure sealants and its effect on their Children's Oral Health. J Clin Pediatr Dent.

[24] Walker KK, Martinez-Mier EA, Soto-Rojas AE, Jackson RD, Stelzner SM, Galvez LC, Smith GJ, Acevedo M, Dandelet L, Vega D (2017). Midwestern Latino caregivers' knowledge, attitudes and sense making of the oral health etiology, prevention and barriers that inhibit their children's oral health: a CBPR approach. BMC Oral Health.

[25] Folayan MO, Kolawole KA, Oyedele T, Chukwumah NM, Onyejaka N, Agbaje H, Oziegbe EO, Oshomoji OV (2014). Association between knowledge of caries preventive practices, preventive oral health habits of parents and children and caries experience in children resident in sub-urban Nigeria. BMC Oral Health.

[26] Schwendicke F, Dorfer CE, Schlattmann P, Foster Page L, Thomson WM, Paris S (2015). Socioeconomic inequality and caries: a systematic review and meta-analysis. J Dent Res.

[27] Van den Branden S, Van den Broucke S, Leroy R, Declerck D, Hoppenbrouwers K (2013). Oral health and oral health-related behaviour in preschool children: evidence for a social gradient. Eur J Pediatr.

[28] Franzman MR, Levy SM, Warren JJ, Broffitt B (2004). Tooth-brushing and dentifrice use among children ages 6 to 60 months. Pediatr Dent.

[29] Nunez L, Icaza G, Contreras V, Correa G, Canales T, Mejia G, Oxman-Martinez J, Moreau J (2013). Factors associated with dental consultation in children in Talca (Chile) and in Chilean immigrants in Montreal (Canada). Gac Sanit.

[30] Camargo MB, Barros AJ, Frazao P, Matijasevich A, Santos IS, Peres MA, Peres KG (2012). Predictors of dental visits for routine check-ups and for the resolution of problems among preschool children. Rev Saude Publica.

[31] Al Agili DE, Griffin SO (2015). Effect of family income on the relationship between parental education and sealant prevalence, National Health and nutrition examination survey, 2005-2010. Prev Chronic Dis.

[32] Bhatt S, Gaur A (2019). Dental caries experience and utilization of Oral Health services among Tibetan refugee-background children in Paonta sahib, Himachal Pradesh, India. J Immigr Minor Health.

[33] Souza JGS, Sampaio AA, Costa Oliveira BE, Jones KM, Martins A (2018). Socioeconomic inequalities in the use of dental care services during early childhood: an epidemiological survey. Int J Paediatr Dent.

[34] Medina-Solis CE, Avila-Burgos L, Marquez-Corona ML, Medina-Solis JJ, Lucas-Rincon SE, Borges-Yanez SA, Fernandez-Barrera MA, Pontigo-Loyola AP, Maupome G. Out-Of-Pocket Expenditures on Dental Care for Schoolchildren Aged 6 to 12 Years: A Cross-Sectional Estimate in a Less-Developed Country Setting. Int J Environ Res Public Health. 2019;16(11):1997. 10.3390/ijerph16111997 PMC6603907 31195612

