# Supplementary material for: Are parents’ education levels associated with either their oral health knowledge or their children’s oral health behaviors? A survey of 8446 families in Wuhan
Source: BMC Oral Health. 2020 Jul 11;20:203. doi: 10.1186/s12903-020-01186-4 (PMC7353758; doi:10.1186/s12903-020-01186-4)
Supplement: Supplementary file 1 — Additional file 1. Questionnaire for Wuhan Hongshan District Primary School Students’ Families. [file 12903_2020_1186_MOESM1_ESM.docx]

1. How many children do you have?

A one child

B two or more children

2. What is the highest educational level of the child’s father?

A Middle school degree or below

B High school degree

C College degree

D Undergraduate degree or above

3. What is the highest educational level of the child’s mother?

A Middle school degree or below

B High school degree

C College degree

D Undergraduate degree or above

4. Does the child usually brush his/her teeth?

A Yes

B No

5. Does the child brush his/her teeth at least twice a day?

A Yes

B No

6. Has the child ever visited a dentist?

A Yes

B No

7. Was the last dental visit within the past 12 months?

A Yes

B No

8. The main reason for the last dental visit.

A Treatment

B Consultation

9 Do you think the following statement is correct?

(1) Gingival bleeding is normal when brushing teeth.

A Correct

B Wrong / do not know

(2) Gingivitis is caused by bacterial infection.

A Correct

B Wrong / do not know

(3) Tooth-brushing is helpful in preventing gingivitis.

A Correct

B Wrong / do not know

(4) Dental caries is mainly caused by pathogenic bacteria.

A Correct

B Wrong / do not know

(5) Sugar intake is associated with dental caries.

A Correct

B Wrong / do not know

(6) Fluoride protects teeth from decay.

A Correct

B Wrong / do not know

(7) Pit and fissure sealants help in preventing dental caries.

A Correct

B Wrong / do not know

(8) Oral health is essential to general health.

A Correct

B Wrong / do not know

10 Have you ever heard of PFS?

A Yes

B No

11 Where would you prefer to have PFS?

A dental hospital

B school

12 What are the determinants of choosing PFS location?

A distance to treatment sites

B level of cross-infection control

C completeness of medical facilities

D effectiveness of emergency response

E children’s willingness

F other reasons
